# Identification of transport systems involved in eflornithine delivery across the blood-brain barrier

**DOI:** 10.3389/fddev.2023.1113493

**Published:** 2023-05-23

**Authors:** Christopher P. Watson, Gayathri Nair Sekhar, Sarah A. Thomas

**Affiliations:** King’s College London, Institute of Pharmaceutical Science, London, United Kingdom

**Keywords:** eflornithine, blood-brain barrier, amino acids, trypanosomiasis, y^+^ system, transporter, OCT, human African trypanosomiasis

## Abstract

Human African Trypanosomiasis (HAT) is a neglected parasitic disease that continues to persist in sub-Saharan Africa. It is fatal if untreated. The first stage of the disease is associated with the presence of the parasite in the periphery and the second stage with the presence of the parasites in the CNS. The treatment of CNS stage HAT requires the drugs to cross the blood-brain barrier (BBB). Eflornithine is an amino acid analogue that is used to treat second stage HAT gambiense both alone and in combination with nifurtimox. Recent studies have identified that accumulation of eflornithine into the parasites (trypanosomes) involves the amino acid transporter (*Trypanosoma brucei* AAT6). In this study we tested the hypothesis that eflornithine uses a cationic amino acid transport system to cross the BBB. We particularly focused on system y^+^ and system B^0,+^. To do this we utilized specialist databases to compare the physicochemical characteristics of relevant molecules and an *in vitro* model of the BBB to explore the mechanisms of eflornithine delivery into the CNS. Our results confirmed that eflornithine is related to the endogenous amino acid, ornithine. At pH 7.4, eflornithine is predominately (92.39%) a zwitterionic (dipolar) amino acid and ornithine is predominately (99.08%) a cationic (tripolar) amino acid. In addition, the gross charge distribution at pH 7.4 of eflornithine is much smaller (+0.073) than that of ornithine (+0.99). Further results indicated that eflornithine utilized a saturable transport mechanism(s) to cross the hCMEC/D3 cell membranes and that transport was inhibited by the presence of other amino acids including ornithine. Eflornithine transport was also sodium-independent and sensitive to a y^+^ system inhibitor, but not a B^0,+^ system inhibitor. Eflornithine transport was also inhibited by pentamidine, suggestive of transport by organic cation transporters (OCT) which are expressed in this cell line. We confirmed expression of the y^+^ system protein, CAT1, and the B^0,+^ system protein, ATB^0,+^, in the hCMEC/D3 cells. We conclude that eflornithine uses the cationic amino acid transporter, system y^+^, and OCT to cross the BBB. This research highlights the potential of system y^+^ to deliver drugs, including eflornithine, across the BBB to treat brain diseases.

## 1 Introduction

Human African trypanosomiasis (HAT) or sleeping sickness is a parasitic disease affecting sub-Saharan Africa. The first stage of the disease is characterised by the presence of parasites in the blood and lymphatic system and is usually asymptomatic and the second stage (or meningo-encephalitic stage) is associated with parasite entry into the brain and a variety of neurological disturbances. It is fatal if left untreated.

Eflornithine or alpha-difluoromethyl ornithine (DFMO) has anti-trypanosomal activity against HAT caused by infection with the parasite, *Trypanosoma brucei* (*T.b.*) *gambiense* (Bacchi et al., 1980; McCann et al., 1981; van Nieuwenhove et al., 1985; Nightingale, 1991; World Health Organization, 2019). Eflornithine monotherapy to treat CNS stage HAT *gambiense* is administered as 56 intravenous infusions at 100 mg/kg (150 mg/kg for children) every 6 h a day for a total of 14 days (World Health Organization, 2019). The common side effects of eflornithine include diarrhoea, dizziness, headaches, seizures and bone marrow toxicity leading to anaemia, leucopenia and thrombocytopenia which are all generally reversed by administering lower doses of the drug or at the end of treatment (Milord et al., 1992; Blum et al., 2001; Burri and Brun, 2003; Babokhov et al., 2013). Importantly, oral doses (vs. intravenous infusion) and shorter administration times (7 vs. 14 days) are less effective at treating second stage gambiense HAT (Pépin et al., 2000; Na-Bangchang et al., 2004).

The use of nifurtimox-eflornithine combination therapy (NECT) has also been explored. Nifurtimox is a stage 2 drug that is orally active against the gambiense form (World Health Organization, 2019). NECT consists of oral nifurtimox and intravenous eflornithine: nifurtimox 15 mg/kg per day orally in three doses for 10 days; eflornithine 400 mg/kg per day intravenously in two 2-h infusions for 7 days (World Health Organization, 2019). The results showed that although NECT did not offer better cure rates than eflornithine monotherapy, it was safer, simpler to administer (infusion every 12 h for 7 days vs. every 6 h for 14 days) and offered potential prevention from parasite resistance to monotherapy (Priotto et al., 2007; Priotto et al., 2009). NECT is now the first-choice treatment for meningo-encephalitic stage gambiense HAT (World Health Organization, 2019). Eflornithine monotherapy is a second-choice treatment (World Health Organization, 2019).

Investigating how eflornithine and nifurtimox reach the CNS if of special interest if we are to understand their effectiveness in treating second stage HAT and design more efficacious and safer therapies. Our research using *in situ* brain perfusion in healthy and trypanosome-infected mice has suggested that the intensive eflornithine regimen required to cure second stage HAT is due to limited blood-brain barrier (BBB) penetration of eflornithine (Sanderson et al., 2008). Interestingly, this low BBB permeability of eflornithine is not related to efflux by the ABC transporter, P-glycoprotein (Sanderson et al., 2008; Yang et al., 2019). In addition, our studies using *in situ* and *in vitro* models of the BBB have revealed that nifurtimox is able to cross the brain capillary wall well (Jeganathan et al., 2011; Watson et al., 2012).

Eflornithine resistance is thought to be related to the expression of amino acid transporters in parasites. It has been demonstrated that loss of a trypanosome amino acid transporter gene (*TbAAT6* (Tb927.8.5450)) conferred eflornithine resistance in *T. brucei brucei* which causes animal trypanosomiasis (Vincent et al., 2010; Baker et al., 2011). Genome-wide RNAi screens have also provided evidence that eflornithine accumulates into the parasite via amino acid transporter 6 (Baker et al., 2011; Schumann Burkard et al., 2011). Eflornithine is a derivative of ornithine which is a cationic amino acid. Various mammalian transport systems for cationic amino acids (CAA) exist including y^+^, y^+^L, b^0,+^, B^0,+^ and b^+^ systems (Deves and Boyd, 1998; Mann et al., 2003). The term ‘transport system’ calls attention to the concept that a complex of different proteins rather than a single carrier protein mediates a distinct transport activity. Only the y^+^, y^+^L and B^0,+^ systems have been identified at the BBB (Stoll et al., 1993; Sánchez del Pino et al., 1995; O’Kane et al., 2006; Kooijmans et al., 2012). They can also transport neutral amino acids (Supplementary Table S1). The specific transporter proteins involved in each transport activity are listed in Supplementary Table S1.

The differing permeability characteristics of the BBB and the blood-cerebrospinal fluid (CSF) barrier, the influence of blood and interstitial fluid flow dynamics and the contributions of CSF sink to final brain concentrations means that eflornithine transport across the BBB cannot be completely resolved using *in vivo* models. In this present study, we utilized an *in vitro* cell culture method to allow a more focused examination of the specific transport systems involved in eflornithine movement across mammalian membranes and the impact of other drugs (including nifurtimox) on CNS delivery across this interface. We were particularly interested in the hypothesis that eflornithine utilized cationic amino acid transporters to cross the BBB. This current study also used physicochemical characterisation to inform assessment of eflornithine movement across the BBB *in vitro*.

## 2 Materials and methods

### 2.1 Physiochemical characterisation

The molecular weight (MW), the log D at pH 7.4 and the gross charge distribution at pH 7.4 of eflornithine and other molecules was obtained from a chemical properties database, MarvinSketch (version 22.9.0, 2022) (MarvinSketch, 2022). The chemical structures and the percentage distribution of the different microspecies at physiological pH was also examined using this database. The predicted physiological charge of the major microspecies of eflornithine and other molecules was also obtained from another specialist database, DrugBank (Wishart et al., 2018). The information was tabulated and the chemical structures of select microspecies presented.

### 2.2 Materials

The immortalized human cerebral microvessel endothelial cell line (hCMEC/D3) was provided under an academic material transfer agreement [^3^H]eflornithine hydrochloride was synthesized and custom radiolabelled (MW 182.2; 500 mCi/mmol, radiochemical purity 97.6%) by Moravek, CA, United States. [^14^C(U)]sucrose (536 mCi/mmol) was purchased from Moravek Biochemicals. All other chemicals were purchased from Sigma-Aldrich, Dorset, United Kingdom (unless stated). Melarsoprol was a gift from Professor Simon Croft (LSHTM, London, United Kingdom), who had received it from Dr Benedict Blayney [Director of Tropical Neglected Diseases Programmes, Sanofi-Aventis (now Sanofi)].

### 2.3 Cell culture

The hCMEC/D3 cell line originated from human brain tissue obtained following surgical excision of an area from the temporal lobe of an adult female with epilepsy, carried out at Kings College Hospital, London, in accordance with the guidelines of the Local Ethics Committee and research governance guidelines (Weksler et al., 2005). This immortalized cell line is a well-established model of the BBB (Weksler et al., 2013). Our group has also confirmed the BBB phenotype of hCMEC/D3 cells by measuring the expression of the vascular endothelial cell phenotype markers (von Willebrand factor and vascular cell adhesion molecule; VCAM1), the tight junction protein (zonula occludens-1; ZO-1), ABC/SLC transporters including cationic amino acid transporter-1 (CAT-1; SLC7A1) and organic cation transporters (OCT1-3; SLC22A1-A3) (Watson, 2012; Watson et al., 2012; Watson et al., 2016; Sekhar et al., 2017). In this study we performed accumulation assays, which provide a relatively simple and rapid approach for exploring the interaction of eflornithine with transport systems expressed in the plasma membrane of continuous cell lines or primary cell cultures. It is noted that the accumulation assay format is different to the permeability assay format which explores transport across the luminal and abluminal endothelial membranes in series and is suitable for primary cell cultures. In brief, hCMEC/D3 cells were split when they reached 80%–90% confluency and then seeded onto collagen coated (0.1 mg/mL) 96 well plates at a density of 2.5 × 10^4^ cells/cm^2^ (passages 28-35).

#### 2.3.1 Accumulation assay format

For the accumulation assays, cells were grown to 100% confluency (which was reached at 4 days and then left for a further 3 days until experiments (7 days after seeding). Medium was changed every 2–3 days. The accumulation buffer (pH 7.4) consisted of 135 mM NaCl, 10 mM HEPES, 5.4 mM KCl, 1.5 mM CaCl_2_, 1.2 mM MgCl_2_ and 1.1 mM glucose dissolved in distilled water. All experiments included [^3^H]eflornithine (720 nM) and [^14^C]sucrose (927 nM) in the accumulation buffer which was placed on the top of the cell monolayers at time zero. The accumulation of [^3^H]eflornithine and [^14^C]sucrose into the cultured brain endothelial cells was measured at 5 different time points (up to 30 min) in the absence (control group) and presence (test groups) of different molecules (Supplementary Table S1).

Test groups included i) self-inhibition experiments with unlabelled eflornithine at 250 μM and 500 μM ii) cross-competition experiments with the structurally similar amino acid, ornithine (55 μM) iii) cross-competition experiments with 100 μM unlabelled cationic amino acids (including *L*-lysine, *L*-arginine, or asymmetric dimethylarginine (ADMA)) iv) cross-competition experiments with the neutral branched-chain amino acid, *L*-leucine (100 μM) v) inhibitor experiments with the system y^+^ inhibitor, *L*-homoarginine (20 mM) (White et al., 1982; White, 1985; O’Kane et al., 2006; Chafai et al., 2017) or the inhibitor of system B^0,+^, (2-aminobicyclo-(2,2,1)-heptane-2-carboxylic acid (BCH); 4 mM) (van Winkle et al., 1985; Mackenzie et al., 1994; Sloan and Mager, 1999). Interestingly, as well as being an inhibitor of system B^0,+^, BCH is known to competitively inhibit most human large neutral amino acid transporters (Christensen et al., 1969; Kim et al., 2008; Gauthier-Coles et al., 2021). vi) Experiments with a Na^+^Cl^−^ free buffer (to inhibit Na^+^Cl^−^ dependent transport). This Na^+^Cl^−^ free buffer was prepared using 240 mM sucrose, 10 mM HEPES, 4 mM potassium-D-gluconate, 2.8 mM CaSO_4_, 1 mM MgSO_4_ and 1 mM D-glucose in distilled water. vii) In addition, the effect of other anti- HAT drugs on radiolabelled eflornithine accumulation in hCMEC/D3 cells was assessed by further cross-competition experiments using the clinically relevant concentrations of anti-HAT drugs, i.e., nifurtimox (6 μM), pentamidine (10 μM), suramin (150 μM) and melarsoprol (30 μM) (Sanderson et al., 2007; Sanderson et al., 2009; Jeganathan et al., 2011; Watson et al., 2012). Nifurtimox, pentamidine and melarsoprol were dissolved in a stock solution of DMSO before being added to the accumulation buffer. The final DMSO concentration was 0.05%. The control accumulation buffer for these experiments also contained 0.05% DMSO.

After the incubation period, buffer was aspirated, and the wells washed with ice cold phosphate buffered saline (PBS) to remove radiolabelled molecules that were not taken up by the cells and to stop further accumulation. Triton X-100 was then added and the plate was incubated for an hour at 37°C to lyse the cells and to release accumulated [^3^H]eflornithine. 100 μL from each of the wells was then transferred to a vial and scintillation fluid added. Radioactivity (disintegrations per minute, dpm) was measured using a Packard Tri-Carb 2900 TR liquid scintillation counter (Perkin-Elmer, United Kingdom) and results corrected for background dpm. The remaining 100 µL in each well were used to perform a bicinchoninic acid (BCA) protein assay. A range of 2–30 μL mg^−1^ of protein was acceptable.

#### 2.3.2 Expression of results

The radioactivity (dpm) accumulated in the cells can be expressed as a function of the radioactivity in the initial accumulation buffer to calculate the volume of distribution (V_d_; μL/mg of protein) of the [^3^H]eflornithine or [^14^C]sucrose as shown in the equation below.

$$Vd=\frac{dpm\;per\;mg\;ofprotein}{\begin{array}{c} dpm\;per\;µl\;of\;accumulation\;buffer \end{array}}$$

The V_d_ for radiolabelled eflornithine were corrected with the V_d_ for [^14^C]sucrose (marker for non-specific binding) at each time point. The [^14^C]sucrose values are also used to confirm barrier integrity of each monolayer. [^14^C]sucrose is a well established marker of BBB integrity (Kadry et al., 2020).

The V_d_ for [^3^H]eflornithine is a measure of accumulation and is effectively the sum of the [^3^H]eflornithine that has entered the cell minus the [^3^H]eflornithine that has left the cell. A V_d_ is calculated at each time point. To interpret the data it is understood i) that both the rate of [^3^H]eflornithine entry into the cell (i.e., cellular influx) and the rate of radiolabelled eflornithine that leaves the cell (i.e., cellular efflux) may be different at each time point. ii) If a test group reduces [^3^H]eflornithine accumulation, an influx transporter is involved. If a test group increases [^3^H]eflornithine accumulation, an efflux transporter is involved. iii) The identity of any influx transporter is more likely to be revealed by studying the earliest time point. iv) An absence of an effect by some test groups may be due to an inhibition of both influx and efflux transporters. v) There may be multiple different types of transporter involved in influx and efflux.

### 2.4 3-(4,5-Dimethylthiazol-2-yl)-2,5-diphenyltetrazolium bromide (MTT) cytotoxicity assay

To ensure the V_d_ results were not affected by any cytotoxic effects of the molecules used in this study, we performed a 3-(4,5-dimethylthiazol-2-yl)-2,5-diphenyltetrazolium bromide (MTT) assay on confluent monolayers of hCMEC/D3 cells after they had been exposed to the various test molecules. This is a colorimetric assay where MTT, a yellow tetrazol, will reduce to form a purple formazan in living cells. This colour change can be measured spectrophotometrically and can be used to confirm cell viability (Denizot and Lang, 1986). Monolayers of cells, which had not been exposed to the test molecules, were used as controls.

### 2.5 SDS page and western blot

Western blots were performed as follows: hCMEC/D3 cells were grown to confluence in T-75 flasks (Thermo Scientific, United Kingdom) and left for 3–4 days. The flask was then transferred to ice and the medium removed. The cells were then washed twice using ice-cold PBS. Then, 1 mL of ice-cold radio immunoprecipitation assay buffer (Sigma-Aldrich, Dorset, United Kingdom) containing protease inhibitors (10% v/v) (Thermo Scientific, Loughborough, United Kingdom) was added to the flask to lyse the cells. The cell lysate was then transferred to a pre-cooled 1.5 mL Eppendorf tube and left on ice for 20 min. The tubes were then centrifuged at 10,000 rpm for 10 min at 4°C using a Thermo Electron Corporation Heraeus Fresco17 bench-top micro-centrifuge. After centrifugation, the supernatant was transferred to another pre-cooled 1.5 mL Eppendorf and the pellet discarded. The resulting supernatant was taken for Western blot analysis to assess the expression of the transporters, CAT-1 (SLC7A1) and ATB^0,+^ (SLC6A14) in the cell line. The protein content of the cell lysate was determined using a BCA assay. Samples (30 μg of protein) were then prepared for SDS -PAGE and the proteins separated using NOVEX 4%-20% Tris-glycine mini protein gels (1.0 mm, 12 well) and the XCell SureLockTM Mini-Cell electrophoresis system (Life Technologies, Paisley, United Kingdom). The gel was run at 160 V for 1 h 20 min. After SDS-page, the gels were placed in transfer buffer (Novex Tris-Glycine Transfer Buffer from Thermo Fisher Scientific, Paisley, United Kingdom) for 10 min. The transfer was then run at 100 V for an hour. After the transfer, the membrane was removed and placed in blocking buffer consisting of 5% non-fat dry milk powder (Tesco, United Kingdom) in PBS-Tween (PBS-T, VWR International Ltd., Leicestershire, United Kingdom) on an orbital shaker for an hour to prevent non-specific binding. After blocking, the membrane was washed twice with PBS-T and incubated with the primary antibody for CAT-1 or the primary antibody for ATB^0,+^ in PBS-T with 5% bovine serum albumin solution (Sigma-Aldrich, Dorset, United Kingdom) overnight at 4°C under constant agitation. The primary antibody for CAT 1 was Rabbit polyclonal (human and mouse) CAT antibody from Abcam (Cat#ab37588; RRID:AB_2190720: dilution 1:250) and for ATB^0,+^ was Rabbit polyclonal SLC6A14 antibody (human and mouse) from Abcam (Cat#ab99102; RRID:AB_10696963: dilution 1:2000). The primary antibody PBS-T with 5% bovine serum albumin solution was then removed, the membrane washed in PBS-T and then incubated with the secondary antibody (Goat anti-rabbit IgG HRP conjugated Abcam ab6721: RRID:AB_955447, dilution 1:2000) for 1 h at room temperature.

The membrane was then washed again with PBS-T three times (10 min each) to remove any unbound antibody. The membrane was incubated with the enhanced chemiluminescence working solution (SuperSignal West Pico Chemiluminescent Substrate, Thermo Fisher Scientific United Kingdom Ltd., Paisley, United Kingdom) for 1 minute and then visualised using enhanced chemiluminescence using Genesnap G:box and software (Syngene) to visualise the protein bands (Ramirez et al., 2009; Rochfort et al., 2014).

Since CAT-1 and ATB^0,+^ can be in their glycosylated forms, N-linked-glycopeptide-(N-acetyl-beta-D-glucosaminyl)-L-asparagine amidohydrolase (PNGaseF) enzyme was used to deglycosylate the proteins. PNGaseF was purchased from New England Biolabs, Hertfordshire, United Kingdom (P0704S). Human liver hepatocellular carcinoma cell (HepG2) lysates or the human wild-type MCF-7 cell lysates were used as a positive control. Samples containing 30 μg of protein were loaded into each lane.

### 2.6 Immunofluorescence

Immunofluorescence of ATB^0,+^ was performed on hCMEC/D3s cells using a previously described protocol (Watson et al., 2012). Cells were grown on rat-tail collagen type 1 coated glass coverslips and then fixed using 4% formaldehyde in PBS for 10 min at 4°C. The coverslips were then washed three times with PBS and treated for 5 min with 0.1% Triton X-100 in PBS at room temperature. Following this permeabilization step, the coverslips were washed three times in PBS and then non-specific sites were blocked with PBS containing 10% serum, 0.1% Triton X-100 for 30 min at RT. The coverslips were then incubated overnight at 4°C with primary antibody (1:500 Rabbit anti-human SLC6A14/ATB^0,+^ polyclonal (Cat#:BMP052 RRID: AB_1953038) MBL International, Caltag-Med Systems Ltd., Buckingham, United Kingdom) in PBS. Following overnight incubation, coverslips were washed three times with PBS and then goat anti-rabbit Alexa Fluor 488 (1:200 in PBS) was added for 1 h at room temperature. Following secondary incubation, coverslips were washed in PBS twice, and incubated in PBS containing 1 μg/mL DAPI nucleus stain (New England Biolabs, Bristol, United Kingdom) for 30 min at room temperature. Coverslips were then washed a final time in PBS, dipped in distilled water and mounted onto slides with PVA-DABCO^®^, before viewing with a Zeiss LSM710 confocal microscope and image analysis software Zen 2009 (Zeiss, Germany).

### 2.7 Data analysis and statistics

Data are all expressed as mean ± SEM unless stated otherwise. Unless stated, data were analysed by one-way ANOVA with Tukey’s *post hoc* test or two-way ANOVA with Holm-Sidak post-hoc test using Sigmaplot version 13 from Systat Software, Inc., San Jose California United States.

## 3 Results

### 3.1 Physicochemical assessment

**FIGURE 1:**
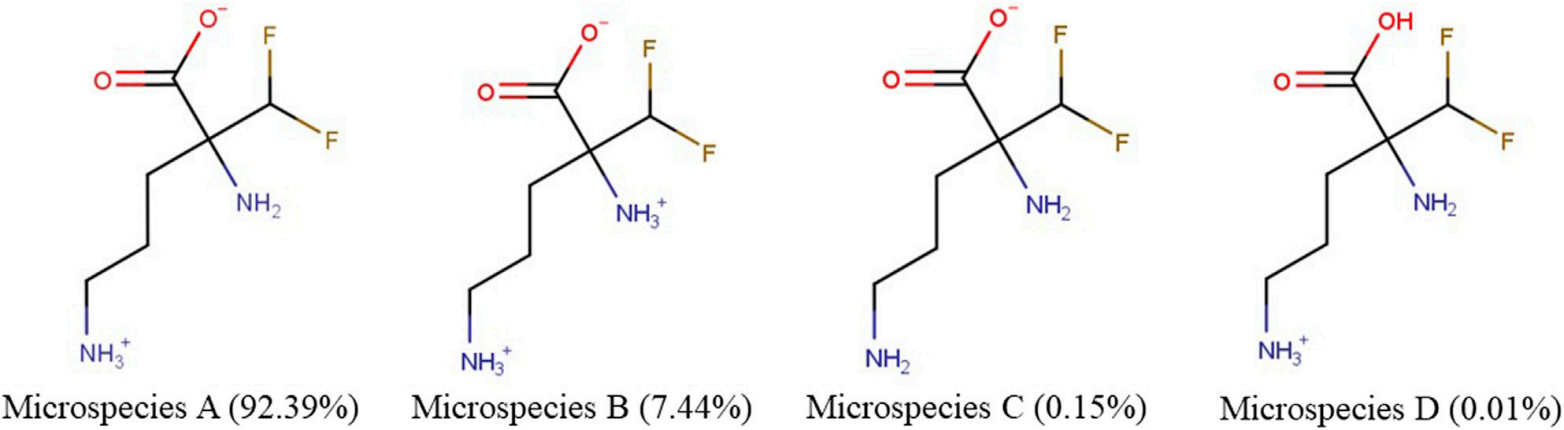
The percentage distribution and chemical structures of the four eflornithine microspecies found at physiological pH.

We examined the physicochemical characteristics of eflornithine and related molecules using chemical property databases including MarvinSketch and DrugBank. Eflornithine has a MW of 182.17 g/mol, a log D of −3.91, a pI of 8.26 and has a gross charge distribution of +0.073 at physiological pH (Table 1). It exists as four microspecies at physiological pH (Figure 1). The predominant (92.39%) microspecies of eflornithine at pH7.4 is a zwitterionic (dipolar) amino acid.

**TABLE 1:** A table listing the physicochemical characteristics for eflornithine and related substances. Unless stated all values were obtained from MarvinSketch (MarvinSketch, 2022). The DrugBank database has been described by Wishart and colleagues (Wishart et al., 2018). The common name, the International Union of Pure and Applied Chemistry (IUPAC) name and the chemical abstracts service (CAS) registry number have been reported for each substance.

Common name	CAS registry number	MW	Gross charge distribution of the molecule at pH 7.4	Physiological charge of major microspecies (DrugBank)	% Distribution of microspecies at pH 7.4 together with their charge
IUPAC name		LogD at pH 7.4			
Eflornithine or 2,5-diamino-2-(difluoromethyl)pentanoic acid	70052-12-9	182.17 −3.91	+0.073	+1	(0) 92.39% (+1) 7.44% (-1) 0.15% (+1) 0.01%
Sucrose or beta-D-arabino-hex-2-ulofuran-osyl alpha-D-gluco-hexopyranoside	57-50-1	342.30 −4.87	−0.00	0	(0) 100%
L-Ornithine or 2,5-diaminopentanoic acid	70-26-8	132.16 −6.01	+0.99	+1	(+1) 99.08% (0) 0.75% (1) 0.16%
L-Arginine or 2-amino-5-carbamimidamidopentanoic acid	74-79-3	174.20 −4.77	+0.98	+1	(+1) 98.15% (0) 1.85%
L-Lysine or 2,6-diaminohexanoic acid	56-87-1	146.19 −5.62	+0.99	+1	(+1) 99.10% (0) 0.74% (0) 0.16%
ADMA or N^5^-(N,N-Dimethyl carbamimidoyl)-L-ornithine	30315-93-6	202.26 −3.99	+0.98	+1	(+1) 98.11% (0) 1.88%
L-Homoarginine or (2S)-2-amino-6-carbamimidamidohexanoic acid	156-86-5	188.23 −4.69	+0.991	+1	(+1) 99.12% (0) 0.87%
L-Leucine or (2S)2amino4methylpentanoic acid	61-90-5	131.17 −2.16	−0.008	0	(0) 99.24% (−1) 0.75%

Eflornithine is a fluoroamino acid that is ornithine substituted by a difluoromethyl group at position 2. Ornithine has a MW of 132.16, a log D of −6.01, a pI of 9.82 and exists as three microspecies at physiological pH (Table 1). The predominant microspecies (99.08%) at physiological pH is positively charged and tripolar (Supplementary Figure S1). Other cationic (tripolar) amino acids such as *L*-arginine, *L*-lysine and ADMA have similar characteristics to ornithine including a gross charge distribution of approximately +0.99 at pH 7.4 (Table 1). The physicochemical characteristics of a neutral amino acid (*L-*leucine) and a system y^+^ inhibitor (*L*-homoarginine) are also reported in Supplementary Figure S1, Table 1. The physicochemical characteristics of the system B^0,+^ inhibitor (BCH) and the anti-HAT drugs, nifurtimox, pentamidine, melarsoprol and suramin, are reported in Supplementary Table S2.

Sucrose is a hydrophilic marker molecule and its physicochemical characteristics are also reported in Table 1. It has a MW of 342.3 and a log D at physiological pH of −4.87. It exists as one neutral microspecies at pH 7.4.

### 3.2 Eflornithine accumulation studies

#### 3.2.1 Time dependence

The volume of distribution (V_d_) of [^3^H]eflornithine and [^14^C]sucrose in the hCMEC/D3 cell line was plotted as a function of time and the line of best fit determined by linear regression analysis. It was assumed that the slope of the line represents the rate of accumulation over 30 min and the ordinate intercept represents the rapidly equilibrating space. The rapidly equilibrating space includes the endothelial cell space and non-specific binding to both the cell membranes and plasticware.

The results are illustrated in Supplementary Figure S2. For [^3^H]eflornithine the rate of accumulation was determined to be 1.3 μL/min/mg of protein over 30 min (*R* ^2^ = 0.89) and the rapidly equilibrating space was determined to be 9.6 μL/mg of protein. For [^14^C]sucrose, the rate of accumulation was 0.1 μL/min/mg of protein over 30 min (*R* ^2^ = 0.91) and the rapidly equilibrating space was determined to be 0.7 μL/mg of protein.

The [^14^C]sucrose V_d_ values act as a negative control and provide baseline values. The baseline values represent non-specific binding to both the cell membranes and plasticware and may also represent passive accumulation of [^14^C]sucrose into the cell. The [^14^C]sucrose V_d_ values were below 6 μL/mg at all time points.

**FIGURE 2:**
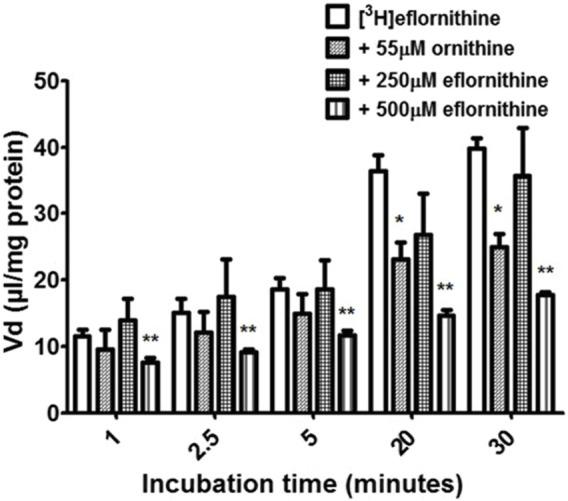
The effect of unlabelled eflornithine and ornithine on the accumulation of [^3^H]eflornithine in hCMEC/D3 cells. All data expressed as mean ± S.E.M, *n* = 3–5 (plates), with 6 replicates (wells) per plate. The [^3^H]eflornithine V_d_ has been corrected for [^14^C]sucrose V_d_. Data were analysed using two-way ANOVA with SigmaPlot 13. Significant differences compared to control (i.e., [^3^H]eflornithine alone) were observed in hCMEC/D3 cells—*p < 0.05, **p < 0.01.

Accumulation of [^3^H]eflornithine was corrected for [^14^C]sucrose (Supplementary Figure S3) and observed to be time dependent (Figure 2). The V_d_ for [^3^ H]eflornithine (corrected for [^14^C]sucrose) at 1 min was approximately 12 μL/mg and significantly increased to approximately 40 μL/mg at 30 min (Unpaired Student’s test; *p* < 0.0001). Figure 2 illustrates that influx of [^3^H]eflornithine is greater than any efflux (if present) of [^3^H]eflornithine at all time points.

#### 3.2.2 Transport mechanism

To determine if there was a saturable mechanism for eflornithine accumulation into the hCMEC/D3 cells, radiolabelled eflornithine was incubated with unlabelled eflornithine (250 μM and 500 μM) and the structurally similar amino acid, ornithine (55 μM). Unlabelled eflornithine (250 μM) did not significantly affect the accumulation of [^3^H]eflornithine (Figure 2). However, there was, on average, a 38% decrease in the accumulation of radiolabelled eflornithine in the presence of unlabelled eflornithine (500 μM) compared to control at all time points (Figure 2) (**p < 0.01). Similarly, in the presence of ornithine, there was significantly decreased accumulation of [^3^H]eflornithine at 20 (36%) and 30 min (37%) (*p < 0.05). No significant differences were found for [^14^C]sucrose between the control and test groups at any time point (Supplementary Figure S3).

#### 3.2.3 Amino acid cross competition

**FIGURE 3:**
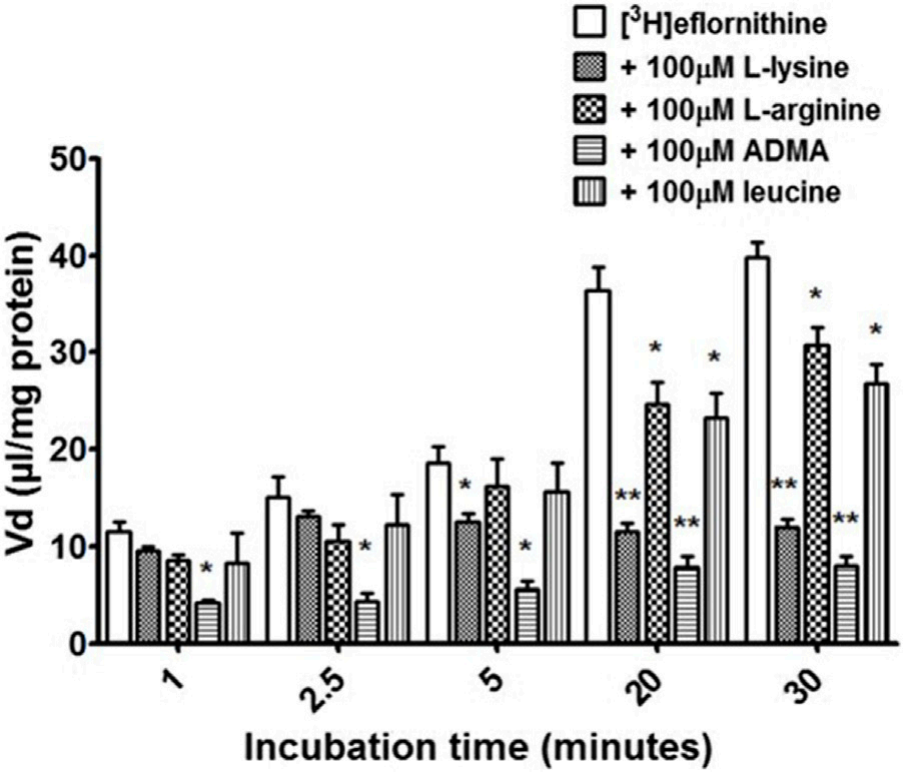
The effect of unlabelled L-lysine, L-arginine, ADMA and leucine on [^3^H]eflornithine accumulation in hCMEC/D3 cells. The [^3^ H]eflornithine V_d_ has been corrected for [^14^C]sucrose V_d_. Significant differences compared to control were observed in hCMEC/D3 cells—*p < 0.05, **p < 0.01. All data expressed as mean ± S.E.M, *n* = 3–4 (plates), with 6 replicates (wells) per plate. Data were analysed using two-way ANOVA with SigmaPlot 13.

Cross-competition studies were performed to explore if eflornithine shared a transport mechanism with other amino acids. In these hCMEC/D3 experiments the amino acids (*L*-lysine, *L*-arginine, ADMA or *L*-leucine) were incubated with [^3^H]eflornithine and [^14^C]sucrose (Figure 3; Supplementary Figure S4).

Radiolabelled eflornithine accumulation significantly decreased in the presence of ADMA (100 μM) at all time points compared to control (decreasing by 64% at 1 min, 71% at 2.5 min, 69% at 5 min, 28% at 20 min and 79% after 30 min Figure 3). *L*-lysine (100 μM) also significantly decreased the accumulation of radiolabelled eflornithine after 5 min (35%; *p* < 0.05), 20 min (68%; *p* < 0.01) and 30 min (69%; *p* < 0.01). Unlabelled *L*-arginine (100 μM) and *L*-leucine (100 μM) significantly decreased [^3^H]eflornithine accumulation at 20 and 30 min (*p < 0.05) (Figure 3). No significant differences were found for [^14^C]sucrose between the control and test groups at any time point (Supplementary Figure S4).

#### 3.2.4 Actions of y^+^ system inhibitors, B^0,+^ system inhibitors and Na^+^-Cl^+^ free buffer

**FIGURE 4:**
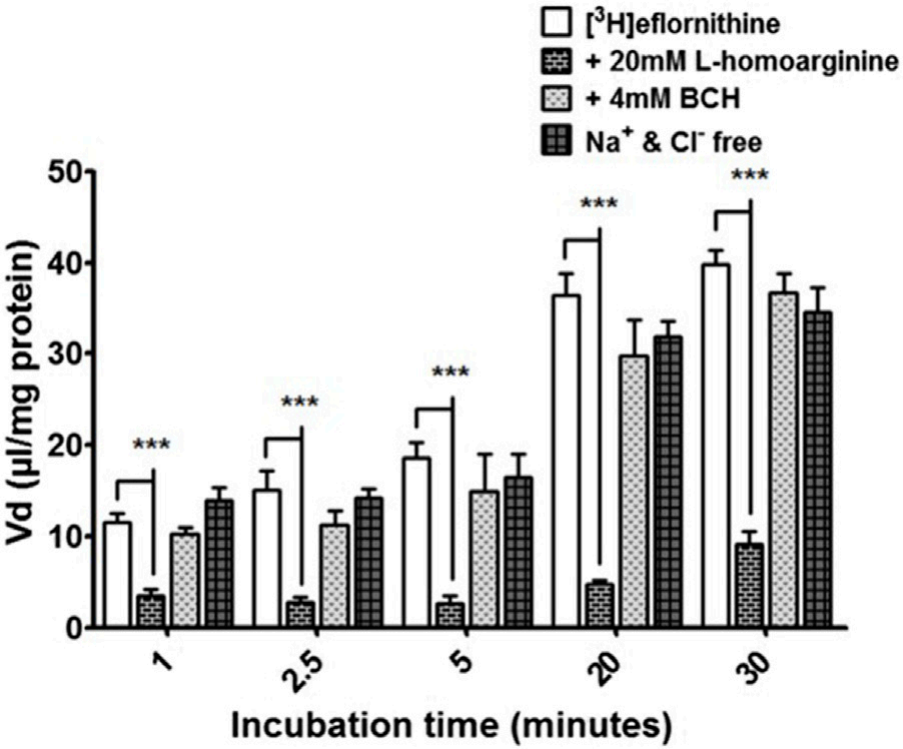
[^3^H]eflornithine accumulation in hCMEC/D3s in the absence and presence of L-homoarginine, BCH or Na^+^Cl^−^ free buffer. The [^3^H]eflornithine V_d_ has been corrected for D-[^14^C]sucrose V_d_. All data expressed as mean ± S.E.M, *n* = 3 (plates), with 6 replicates (wells) per plate. Data were analysed using two-way ANOVA with SigmaPlot 13. Significant differences compared to control were observed with L-homoarginine at all time-points—***p < 0.001.

Radiolabelled eflornithine was incubated with L-homoarginine (inhibitor for system y^+^), BCH (inhibitor of system B^0,+^) or Na^+^Cl^−^ free buffer (to inhibit Na^+^Cl^−^ dependent amino acid transport mechanisms) to assess the potential impact of these test conditions (Figure 4). The accumulation of [^3^H]eflornithine significantly decreased in the presence of *L*-homoarginine (20 mM) compared to control at all time points (80% decrease in accumulation on average, ***p < 0.001). In contrast, BCH or a Na^+^Cl^−^ free buffer did not affect the accumulation of radiolabelled eflornithine in the hCMEC/D3 cell line. No significant differences were found for [^14^C]sucrose between the control and test groups at any time point (Supplementary Figure S5).

#### 3.2.5 Interactions with other anti-HAT drugs

**FIGURE 5:**
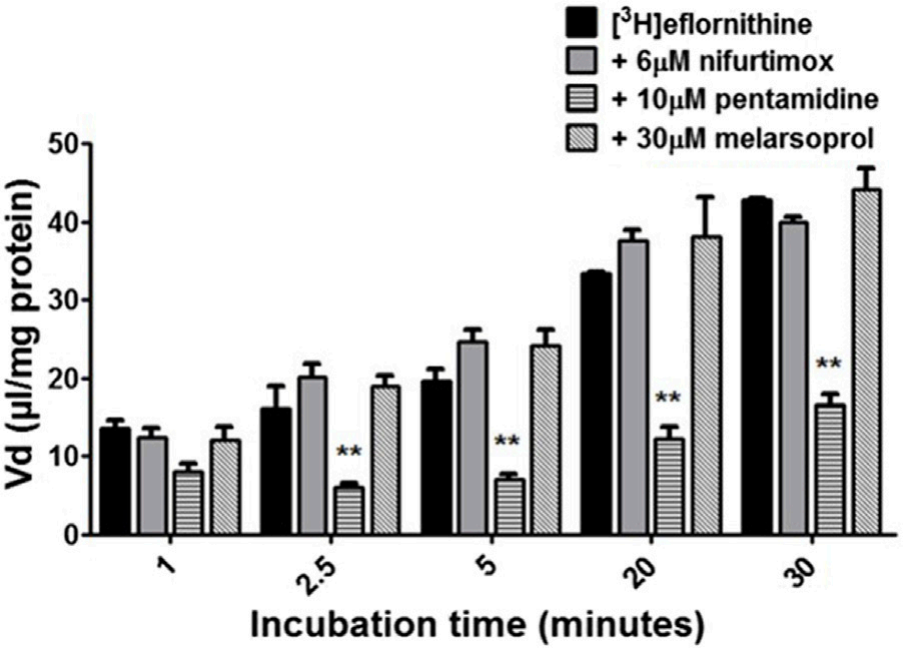
The effect of anti-HAT drugs on radiolabelled eflornithine accumulation in hCMEC/D3 in the presence of DMSO. The [^3^H]eflornithine. V_d_ has been corrected for [^14^C]sucrose V_d_. All data expressed as mean ± S.E.M, *n* = 3 (plates), with 6 replicates (wells) per plate. Data were analysed using two-way ANOVA with SigmaPlot 13. Significant differences were observed with pentamidine in hCMEC/D3 cells -**p < 0.01. The control and test groups were performed in the presence of 0.05% DMSO.

**FIGURE 6:**
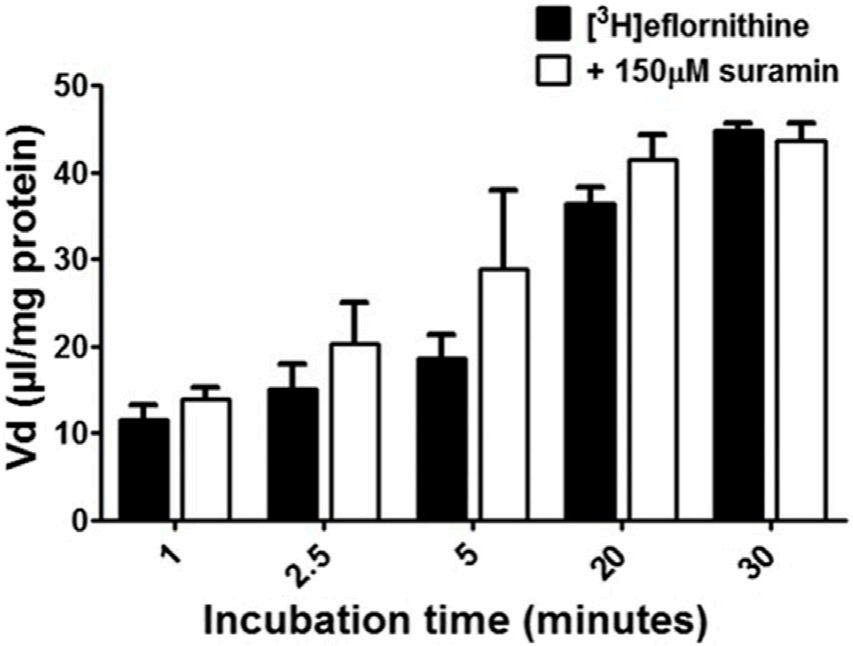
The effect of suramin on [^3^H]eflornithine accumulation in hCMEC/D3s. The [^3^H]eflornithine V_d_ has been corrected for [^14^C]sucrose V_d_. All data expressed as mean ± S.E.M, *n* = 4 plates, with 6 replicates (wells) per plate. Data were analysed using two-way ANOVA with SigmaPlot 13. No differences compared to control were observed.

To understand if there is competition for transport between anti-HAT drugs, radiolabelled eflornithine was incubated with clinically relevant concentrations of nifurtimox (6 μM), pentamidine (10 μM), melarsoprol (30 μM) (Figure 5) or suramin (150 μM) (Figure 6).

To aid solubility of nifurtimox, pentamidine and melarsoprol, their accumulation buffers contained 0.05% DMSO (Figure 5). Their control group buffer also contained 0.05% DMSO. Suramin is more water soluble so did not require DMSO in the accumulation buffer in either the test or control group so the data is presented separately (Figure 6). The accumulation of radiolabelled eflornithine was only significantly decreased in the presence of pentamidine in hCMEC/D3 cells at 2.5, 5, 20 and 30 min (*p* < 0.01; Figures 5, 6). There was an average decrease of 63% at each of these time points. No significant differences were found for [^14^C]sucrose between the control and test groups at any time point (Supplementary Figures S6, S7).

The accumulation of [^3^H]eflornithine and [^14^C]sucrose in the absence or presence of 0.05% DMSO was compared. There was no significant difference in the accumulation of the radiolabelled molecules between the two control groups at any time point.

### 3.3 MTT assay

**FIGURE 7:**
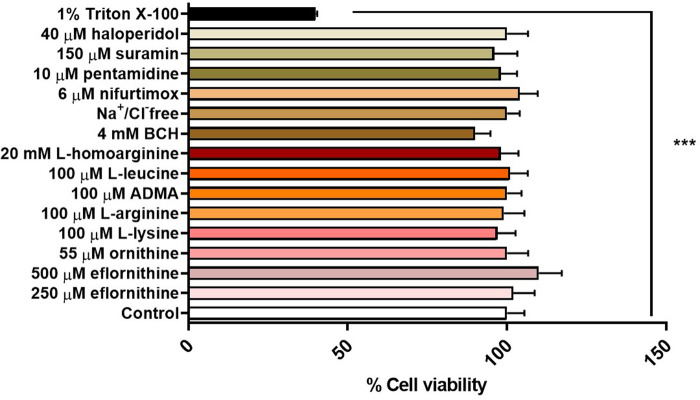
Potential cytotoxic effects of test conditions were assessed by an MTT assay in hCMEC/D3 over 30 min. No significant effects were observed except for the positive control 1% Triton X-100 (***p < 0.001). All data expressed as mean ± S.E.M, *n* = 4 plates, with 6 replicates (wells) per plate. Data were analysed using one-way ANOVA with SigmaPlot.

An MTT assay was performed to confirm absence of cytotoxic effects of the test condition on hCMEC/D3 cells. No cytotoxic effects were observed for any test condition except for the positive control, which was 1% Triton X-100 dissolved in accumulation buffer (Figure 7).

### 3.4 Expression of transporters

#### 3.4.1 CAT-1

**FIGURE 8:**
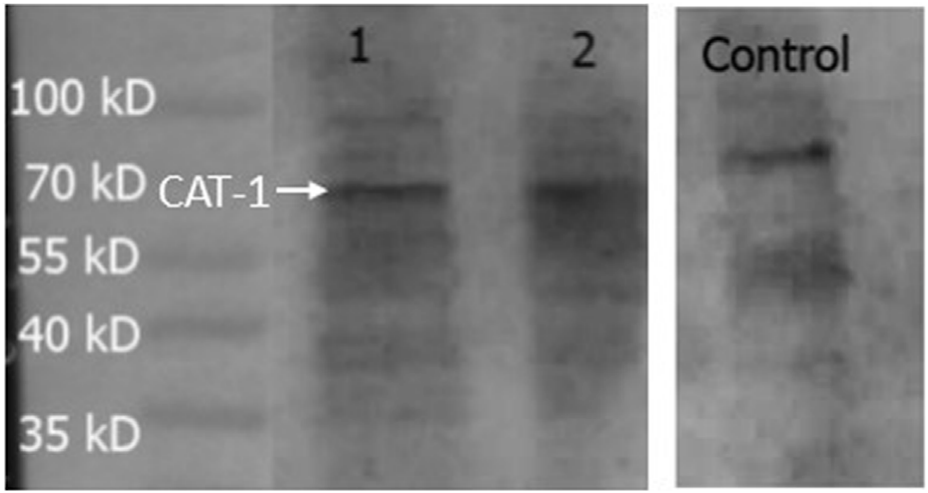
Expression of CAT-1 in hCMEC/D3 cells. HepG2 cells was used as a positive control. A band at 68 kD was observed in all lanes and assumed to be CAT-1. Lane 1- hCMEC/D3 passage 28, Lane 2- hCMEC/D3 passage 33, Lane 3- control HepG2 cells. Lanes 1, 2 and 3 present on same blot.

Expression of CAT-1, a protein that is a member of system y^+^, was measured in the hCMEC/D3 cells (Figure 8). PNGase enzyme was used to deglycosylate the protein after denaturing. CAT-1 expression was found at 68 kD.

#### 3.4.2 ATB^0,+^

**FIGURE 9:**
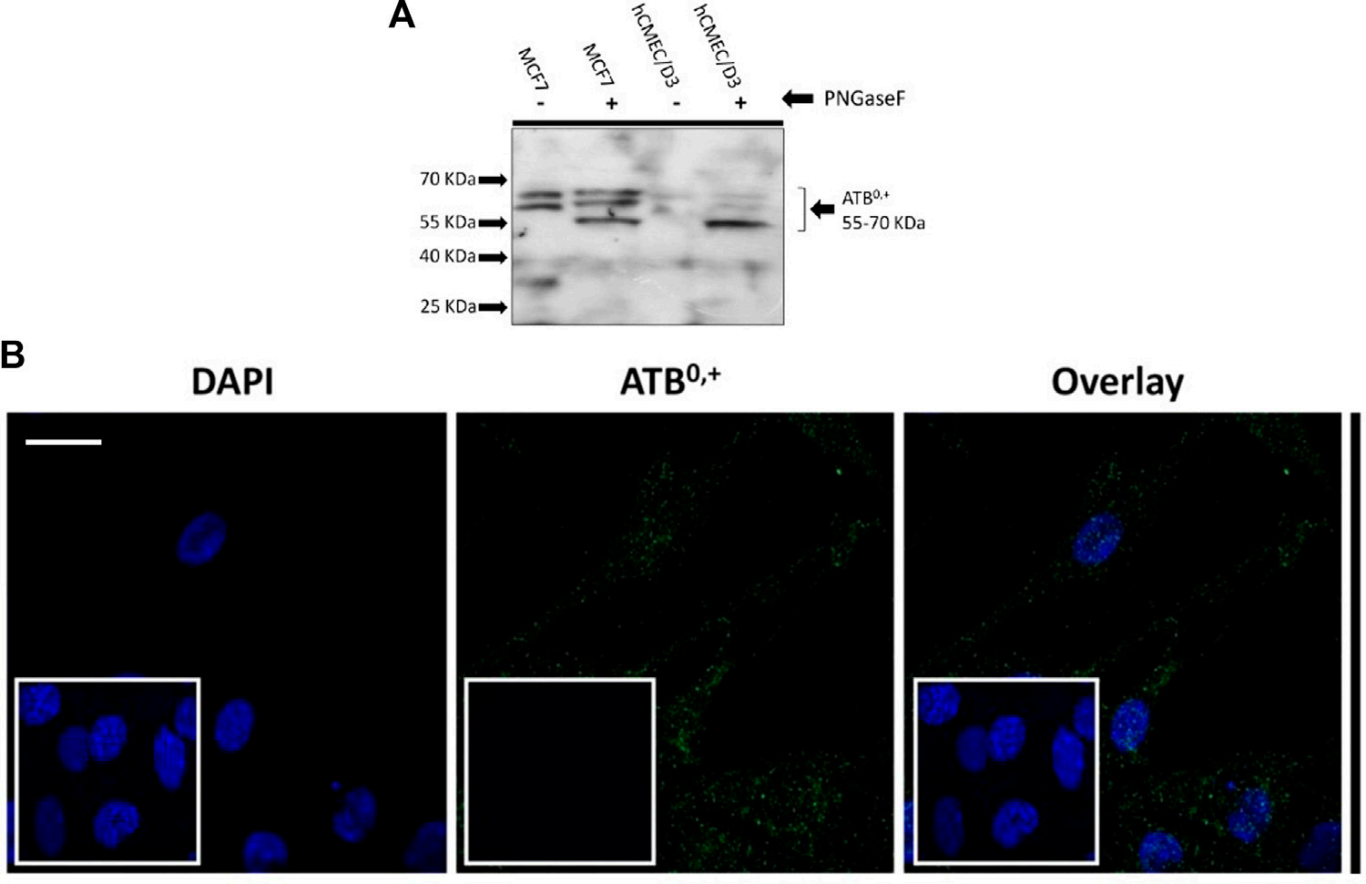
Expression of ATB^0,+^ in hCMEC/D3 cells. **(A)** An example Western blot. Following deglycosylation with PNGase F, SDS-PAGE and WB analysis revealed ATB ^0,+^ expression in hCMEC/D3 (passage 28) and wild type MCF7 whole cell lysate lysed in TGN lysis buffer. Bands from 70 to 55kD were observed. Lane 1 –MCF7 without PNGase, Lane 2-MCF7 with PNGase, Lane 3- hCMEC/D3 passage 28 without PNGase, Lane 4-hCMEC/D3 passage 28 with PNGase. **(B)** ATB ^0,+^ expression was also demonstrated by immunofluorescence performed on hCMEC/D3 cells (passage 30) grown on rat tail collagen type-1-coated coverslips, fixed with 4% formaldehyde and stained for the primary and secondary antibody and viewed at 63x with oil emersion using a Zeiss LSM710 confocal microscope and image analysis software Zen 2009. Scale bar 10 μm. Cell nuclei were counterstained with 1 mg/mL DAPI. For negative staining, cells were stained with secondary antibody only along with DAPI (inset figures).

Expression of the amino acid transporter protein, ATB^0,+^ was studied in the hCMEC/D3 cells. The cell lysates were treated with PNGase enzyme to deglycosylate the protein. Bands were visible from 70-55 kD (Figure 9A). The expression and localisation of ATB^0,+^ was also visualised by immunofluorescence using the rabbit anti-human ATB^0,+^ primary antibody and goat anti-rabbit Alexa Fluor 488 conjugated secondary antibody with confocal microscopy (Figure 9B).

## 4 Discussion

The prospect of survival for patients suffering HAT is reduced if diagnosis is not made during stage 1 of the disease. This is because during stage 2, the parasites enter the brain and are effectively protected from the action of trypanocidal drugs by the BBB. To kill the parasites in the brain, sufficient concentrations of the drugs must be able to cross the BBB. Stage 2 drugs are more likely to cause adverse events than stage 1 drugs. Treatment of HAT is therefore stage specific.

NECT is the first line treatment for stage 2 HAT gambiense and eflornithine monotherapy is a second-line treatment for stage 2 HAT gambiense (World Health Organization, 2019). A clearer understanding of the specific transport pathways for these drugs across the BBB is required if we are to design more effective and safer drugs and drug combinations. In this present study we utilized an *in vitro* model of the human BBB (hCMEC/D3) to investigate the mechanisms of eflornithine delivery to the brain. We were particularly interested in the hypothesis that eflornithine utilized transporters for amino acids to cross the BBB as there is evidence that eflornithine uses an amino acid transporter to accumulate into the blood-stream form of *T. brucei* (Baker et al., 2011).

Our initial investigations utilized specialist databases to explore the physicochemical characteristics of eflornithine and other relevant molecules (Table 1; Supplementary Table S2). Interestingly, there are four microspecies of eflornithine at physiological pH (Figure 1). The predominant eflornithine microspecies (92.39%) is a zwitterionic (dipolar) amino acid. Eflornithine is a structural analogue of the amino acid, ornithine. Three microspecies of ornithine exist at physiological pH-the predominant microspecies (99.08%) is a (tripolar) cationic amino acid (Supplementary Figure S1). Eflornithine has a gross charge distribution at physiological pH of +0.07. This is lower than the gross charge distribution at pH7.4 of the cationic amino acids such as L-ornithine (+0.99), L-lysine (+0.99), L-arginine (+0.98) and ADMA (+0.98).

Our cell culture studies measured radiolabelled eflornithine accumulation as a V_d_ at five timepoints over 30 min and determined that [^3^H]eflornithine was able to enter the cells at a greater rate than that measured for the baseline marker molecule, [^14^C]sucrose (1.3 versus 0.1 μL/min/mg of protein) (Supplementary Figure S2). This will be partly related to the smaller molecular weight (182.17 versus 342.3) and greater lipophilicity (log D at pH 7.4 of −3.91 versus −4.87) of eflornithine when compared to sucrose (Table 1). It may also be related to the presence of transporters for eflornithine, but not sucrose (Kadry et al., 2020).

We compared the accumulation of radiolabelled eflornithine in the absence (control group) and presence (test groups) of different molecules at the five time points. Our self-inhibition studies with 250 μM and 500 μM unlabelled eflornithine indicated that there was a low affinity transporter (with an estimated half-saturation constant (K_m_) of approximately 500 μM) for [^3^H]eflornithine entry into the human BBB cells. Interestingly, this transporter was not detectable using eflornithine concentrations between 720 nM and 250 μM in this cellular model and between 1–250 μM in our earlier studies using the *in situ* brain perfusion method in mice (Sanderson et al., 2008). The reproducibility of these *in vitro* and *in situ* results, together with the cost-effectiveness of the cell culture assays (i.e., lower amounts of inhibitor required per experiment compared to *in situ* brain perfusion experiments) and reduction in the number of time-consuming animal studies in line with the 3Rs (replacement, refinement, and reduction) supported the continued use of hCMEC/D3 to study eflornithine transport in more detail. Based on our physicochemical analyses we focused our pharmacokinetic studies on transporters which are expressed at the BBB and are able to transport cationic and neutral amino acids (Supplementary Table S1).

The influx transporter for eflornithine identified *in vitro* was inhibitable by the cationic amino acids such as *L*-ornithine, *L*-lysine, *L*-arginine, ADMA, as well as the neutral amino acid, *L*-leucine. Selective inhibitor studies revealed that the transporter for [^3^H]eflornithine was sensitive to L-homoarginine (a system y^+^ inhibitor) with a 80% decrease in influx, and did not require the presence of sodium in the accumulation buffer.

Interestingly, system y^+^ is known to accept some zwitterionic amino acids (such as *L*-glutamine and *L*-homoserine) as weak substrates in the presence, but not in the absence, of sodium (White et al., 1982). The gross charge distribution at pH 7.4 for *L*-glutamine is −0.01 and for *L*-homoserine is −0.02 (MarvinSketch, 2022). It has been suggested that the Na^+^ takes the place of the positively charged side chain of cationic (tripolar) amino acids at y^+^- binding sites and so allows the transport of these zwitterionic (dipolar) amino acids (Christensen, 1984; van Winkle et al., 1988). It may be that the gross charge distribution at physiological pH for eflornithine being positive (i.e., +0.073) allows transport of this particular zwitterion by system y^+^ to be independent of sodium.

The transporter proteins, CAT1, CAT2B and CAT3, exhibit system y^+^ activity and the predominant system y^+^ protein at the BBB is CAT1(Stoll et al., 1993) (Smith, 2000). We have previously identified the presence of CAT1 in hCMEC/D3 cells using Western blotting and immunofluorescence (Watson et al., 2016). This was also confirmed in this present study using Western blot. CAT1 is a uniporter involved in loading cells with amino acids down their concentration gradient (Gauthier-Coles et al., 2021). Interestingly, *L*-homoarginine is a substrate of the human cationic amino acid transporters, CAT1, CAT2A and CAT2B and its cellular uptake has been shown to be inhibited by ADMA and *L*-arginine (Chafai et al., 2017). Thus the decrease in [^3^H]eflornithine accumulation in the presence of *L*-homoarginine, ADMA or *L*-arginine in this present study further indicates system y^+^ (CAT) involvement in the transport of eflornithine. The sensitivity of [^3^H]eflornithine accumulation into the hCMEC/D3 cells due to the different substrates/inhibitors varied (Figures 2–6). This is likely related to the different affinities of transporter(s) for different substrates. For example, in a human embryonic kidney cell model CAT-1 transports *L*-arginine, ADMA and *L*-homoarginine with an apparent K_m_ of 519, 183 and 175 μM, respectively (Strobel et al., 2012; Chafai et al., 2017).

System B^0,+^ accepts CAAs and zwitterionic amino acids, with transport sensitive to BCH and coupled to 2Na^+^ and 1Cl^-^ (van Winkle et al., 1988; Deves and Boyd, 1998; Sloan and Mager, 1999). However, radiolabelled eflornithine accumulation was unaffected by the presence of BCH and absence of Na^+^Cl^−^, suggesting that eflornithine is not transported by system B^0,+^. The transporter protein for system B^0,+^ is ATB^0,+^ (Sloan and Mager, 1999). ATB^0,+^ is a symporter thought to be involved in the uptake of amino acids into hCMEC/D3 cells (Kooijmans et al., 2012; Gauthier-Coles et al., 2021). The substrate profile of ATB^0,+^ is debatable with evidence that arginine (and lysine) transport by this protein is possibly not physiologically relevant (Ahmadi et al., 2018; Fairweather et al., 2021). Although, ATB^0,+^ has significant potential as a delivery system for amino acid-based drugs and prodrugs (Hatanaka et al., 2004; Kooijmans et al., 2012) our study indicates that it is unlikely to transport eflornithine. Our Western blot and immunofluorescence studies in hCMEC/D3 cells demonstrated the presence of ATB^0,+^ (SLC6A14). ATB^0,+^ expression in hCMEC/D3 cells has previously been observed (Kooijmans et al., 2012). ATB^0,+^ typically has both membrane and cytoskeletal patterning and has a polarised expression being found at the luminal membrane of bovine brain capillary endothelial cells (Czeredys et al., 2008).

Together our results indicated that [^3^H]eflornithine mainly enters the cell by means of system y^+^ (likely CAT1) and this pathway for eflornithine is sodium-independent. Eflornithine does not utilize system B^0,+^. Eflornithine is likely to also enter the hCMEC/D3 cells by passive diffusion as none of the inhibitor studies completely stopped [^3^H]eflornithine accumulation resulting in a non-saturable component to the accumulation. The log D at physiological pH of eflornithine is higher than all the other positively charged amino acids measured (Table 1), which suggests it would be able to cross membranes by passive diffusion more easily.

Further work was carried out to assess the influence of other anti-HAT drugs, including nifurtimox, on the accumulation of [^3^H]eflornithine into hCMEC/D3 cells. The unlabelled anti-trypanosomal drug concentrations are comparable to those measured in the plasma of treated patients [either 250 μM eflornithine, 150 μM suramin, 200 μM suramin (Milord et al., 1993), 10 μM pentamidine (Waalkes and DeVita, 1970), 30 μM melarsoprol or 6 μM nifurtimox (Gonzalez-Martin et al., 1992)]. Importantly for NECT therapy, unlabelled nifurtimox did not affect the cellular delivery of radiolabelled eflornithine. This was also observed in our *in situ* brain perfusion experiments in mice (Sanderson et al., 2008). In hCMEC/D3 cells, only pentamidine was found to significantly decrease (by approximately 63%) the accumulation of radiolabelled eflornithine suggesting a common transporter for eflornithine and pentamidine. Pentamidine is positively charged at physiological pH (+2; Supplementary Table S2; DrugBank (Wishart et al., 2018)) and has been found to utilize OCT1 to cross the BBB (Sekhar et al., 2017). Interestingly, our earlier hCMEC/D3 investigations with an inhibitor of OCT, haloperidol, also indicated that eflornithine may be transported by OCT at the BBB with transport significantly decreasing by approximately 11% at 2 h (Sekhar et al., 2019).

Early parasite studies could not demonstrate saturable transport of eflornithine into *T. brucei brucei* between the concentrations of 5 μM–10 mM. This suggests eflornithine accumulation was by passive diffusion (Bellofatto et al., 1987) and/or possibly involved a very high affinity (i.e., K_m_ <5 μM) transporter (Bitonti et al., 1986). Other studies have revealed that eflornithine uptake into *T. brucei* is temperature dependent and can be absent in mutant strains (Phillips and Wang, 1987)—suggestive of transporter activity. More recently, studies have identified the amino acid transporter 6 (AAT6) as transferring eflornithine into *T. brucei (T.b.)* (Vincent et al., 2010; Baker et al., 2011; Schumann Burkard et al., 2011) (Mathieu et al., 2014). TbAAT6 is a member of the amino acid/auxin permease (AAAP: TC#2.A.18) family and has been shown to be a low affinity and low-selective transporter for neutral amino acids including proline and glycine (Mathieu et al., 2014). The gross charge distribution at pH 7.4 for proline is −0.001 and for glycine is −0.014 (MarvinSketch, 2022). Other members of the AAAP family expressed in *T. brucei* (*Tb*) have now been characterized (Mathieu et al., 2017). They have a high affinity and are highly selective for a specific positively charged amino acid. For example, TbAAT5-3 transports arginine, TbAAT16-1 transports lysine, TbAAT10-1 transports ornithine and TbAAT2-4 transports ornithine (Macedo et al., 2017). Other Trypanosomatidae also express selective AAAP amino acid transporters. For example, *Trypanosoma cruzi* (*Tc*) express TcAAAP411 and TcCAT1.1 (Carrillo et al., 2010; Henriques et al., 2015) and *Leishmania donovani* (*Ld*) express LdAAP (Shaked-Mishan et al., 2006). They have all been characterized as high-affinity arginine transporters. Lysine transporters have also been identified (TcAAP7 and LdAAP7) (Miranda et al., 2012).

The transfer of cationic amino acids by different mono-specific/highly selective transporters in parasites, contrasts with the less selective mammalian cationic amino acid transporters which transfer several different cationic amino acids (Supplementary Table S1). These latter transporters are members of the SLC7 family also referred to as the APC (amino acid-polyamine organocation: TC #2.A.3) transporter family. Interestingly, one member of the APC family has been identified in the *T. brucei* genome (Berriman et al., 2005; Mathieu et al., 2014).

The interaction of eflornithine with mammalian transporters has proven difficult to resolve using *in vivo* methods (Sanderson et al., 2008). Interestingly, this current physicochemical assessment and *in vitro* study suggests that eflornithine is a substrate for mammalian transporters–in particular the cationic amino acid transporter, system y+, and OCTs. It is likely that these transporters mediate eflornithine delivery across the BBB in sufficient concentrations to treat meningo-encephalitic stage gambiense HAT**.** This research indicates that the cationic amino acid transporter, system y^+^, may be utilized by drugs to cross the BBB. It therefore has potential as a target for drug candidates that need to reach the CNS.

## Data Availability

The original contributions presented in the study are included in the article/Supplementary Material, further inquiries can be directed to the corresponding author.

## Supplementary material

SUPPLEMENTARY FIGURE S1 The chemical structure of the major microspecies of eflornithine and other amino acids at physiological pH.

SUPPLEMENTARY FIGURE S2 Line graphs showing the volume of distribution of [^3^H]eflornithine and [^14^C]sucrose into hCMEC/D3 cells plotted as a function of incubation time. The V_d_ of [^3^H]eflornithine has not been corrected for [^14^C]sucrose. All data expressed as mean ± S.E.M, *n* = 3–4 (plates), with 6 replicates (wells) per plate. Linear regression analysis of the data determined the lines of best fit which are shown as dashed lines. The rate of accumulation over 30 min can be determined as the slope (m) of the computed regression lines and is reported together with the ordinate intercept (c) and the coefficient of determination or R^2^ -value. The ordinate intercept represents the rapidly equilibrating space which includes the endothelial cell space and non-specific binding to both the cell membranes and plasticware.

SUPPLEMENTARY FIGURE S3 The effect of unlabelled eflornithine and ornithine on the accumulation of [^14^C]sucrose in hCMEC/D3 cells. All data expressed as mean ± S.E.M, *n* = 3–5 (plates), with 6 replicates (wells) per plate. Data were analysed using two-way ANOVA with SigmaPlot 13. No significant differences compared to control were observed.

SUPPLEMENTARY FIGURE S4 The effect of unlabelled L-lysine, L-arginine, ADMA or leucine on [^14^C]sucrose accumulation in hCMEC/D3 cells. Significant differences compared to control were not observed in hCMEC/D3 cells. All data expressed as mean ± S.E.M, *n* = 3–4 (plates), with 6 replicates (wells) per plate. Data were analysed using two-way ANOVA with SigmaPlot 13.

SUPPLEMENTARY FIGURE S5 [^14^C]sucrose accumulation in hCMEC/D3s in the absence and presence of L-homoarginine, BCH or Na^+^Cl^-^ free buffer. All data expressed as mean ± S.E.M, *n* = 3 (plates), with 6 replicates (wells) per plate. Data were analysed using two-way ANOVA with SigmaPlot 13. No significant differences compared to control were observed at all time-points.

SUPPLEMENTARY FIGURE S6 The effect of anti-HAT drugs on [^14^C]sucrose accumulation in hCMEC/D3 in the presence of DMSO. All data expressed as mean ± S.E.M, *n* = 3 (plates), with 6 replicates (wells) per plate. Data were analysed using two-way ANOVA with SigmaPlot 13. No significant differences were observed in hCMEC/D3 cells. The control and test groups were performed in the presence of 0.05% DMSO.

SUPPLEMENTARY FIGURE S7 The effect of suramin on [^14^C]sucrose accumulation in hCMEC/D3s. All data expressed as mean ± S.E.M, *n* = 4 plates, with 6 replicates (wells) per plate. Data were analysed using two-way ANOVA with SigmaPlot 13. No differences compared to control were observed.

## Abbreviations

AAAP, amino acid/auxin permease; ADMA, asymmetric dimethylarginine; APC, amino acid-polyamine organocation; BAT, broad-scope amino acid transporter; BBB, blood-brain barrier; BCA, bicinchoninic acid; BCH, 2-aminobicyclo-(2,2,1)-heptane-2-carboxylic acid; BCRP, breast cancer resistance protein; CAA, cationic amino acid; CAT, cationic amino acid transporter; CSF, cerebrospinal fluid; DFMO, eflornithine or alpha-difluoromethyl ornithine; dpm, disintegrations per minute; HAT, human African trypanosomiasis; Hep G2, human liver hepatocellular carcinoma cell line; K_m_, half-saturation constant; *Ld*, *Leishmania donovani*; MW, molecular weight; MTT, (3-(4,5-dimethylthiazol-2-yl)-2,5-diphenyltetrazolium bromide; NAA, neutral amino acid; NECT, nifurtimox-eflornithine combination therapy; OCT, organic cation transporter; PBS, phosphate buffered saline; PBS-T, PBS-Tween; *Tb*, *Trypanosoma brucei*; *Tc*, *Trypanosoma cruzi*; V_d_, the volume of distribution.
