# Supplementary figures and images for: Identification of transport systems involved in eflornithine delivery across the blood-brain barrier
Source: Front Drug Deliv. Author manuscript; Available in PMC 2024 Mar 13. (PMC7615738; doi:10.3389/fddev.2023.1113493)

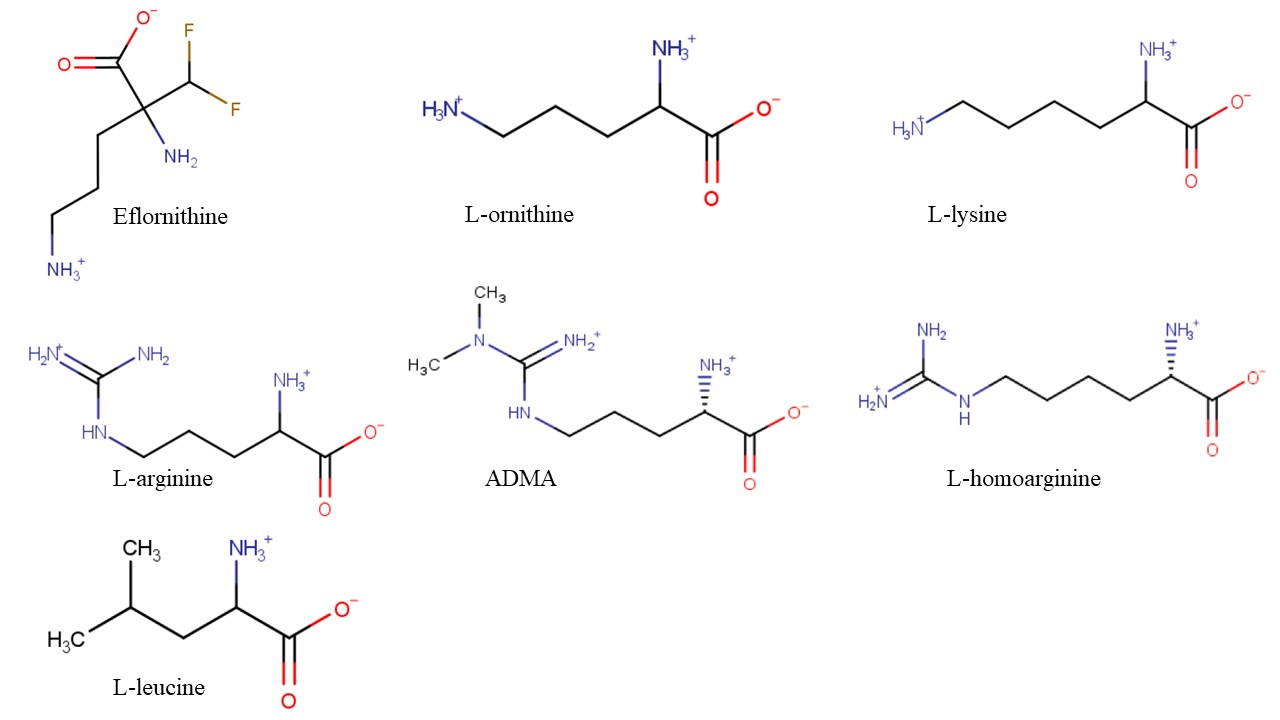

Supplement: Figure S1 [file EMS194562-supplement-Figure_S1.jpg]

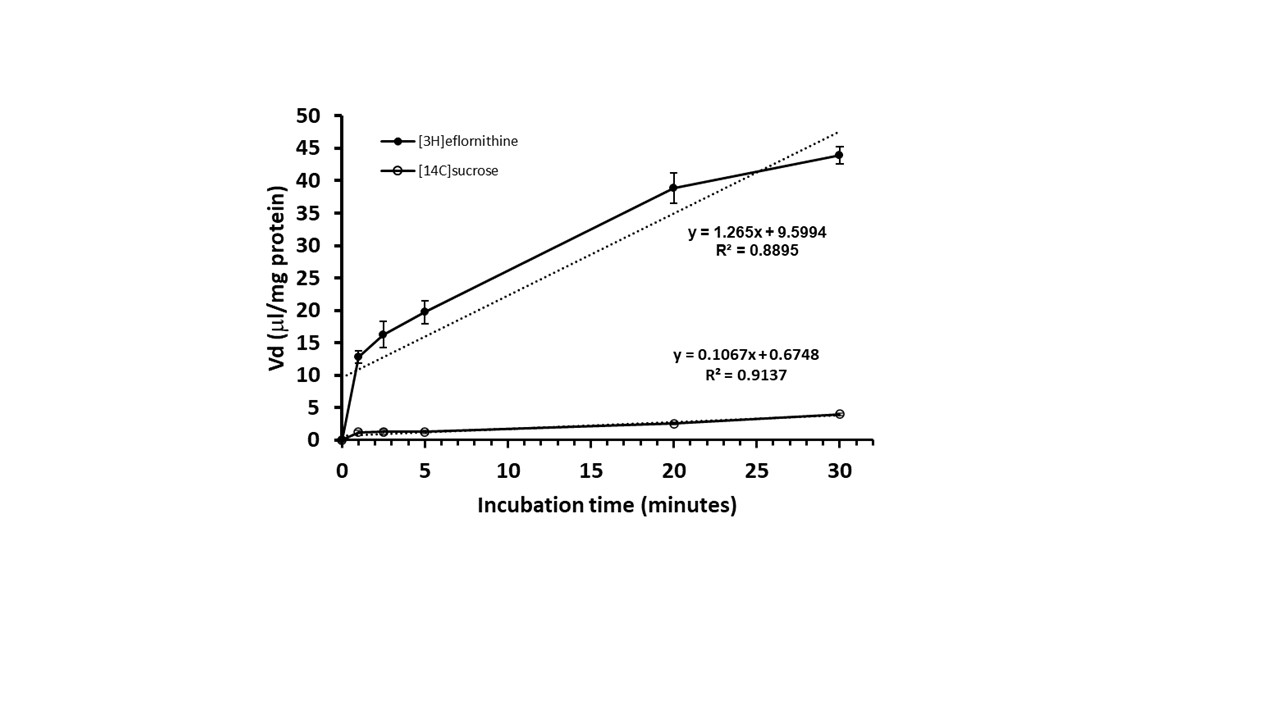

Supplement: Figure S2 [file EMS194562-supplement-Figure_S2.jpg]

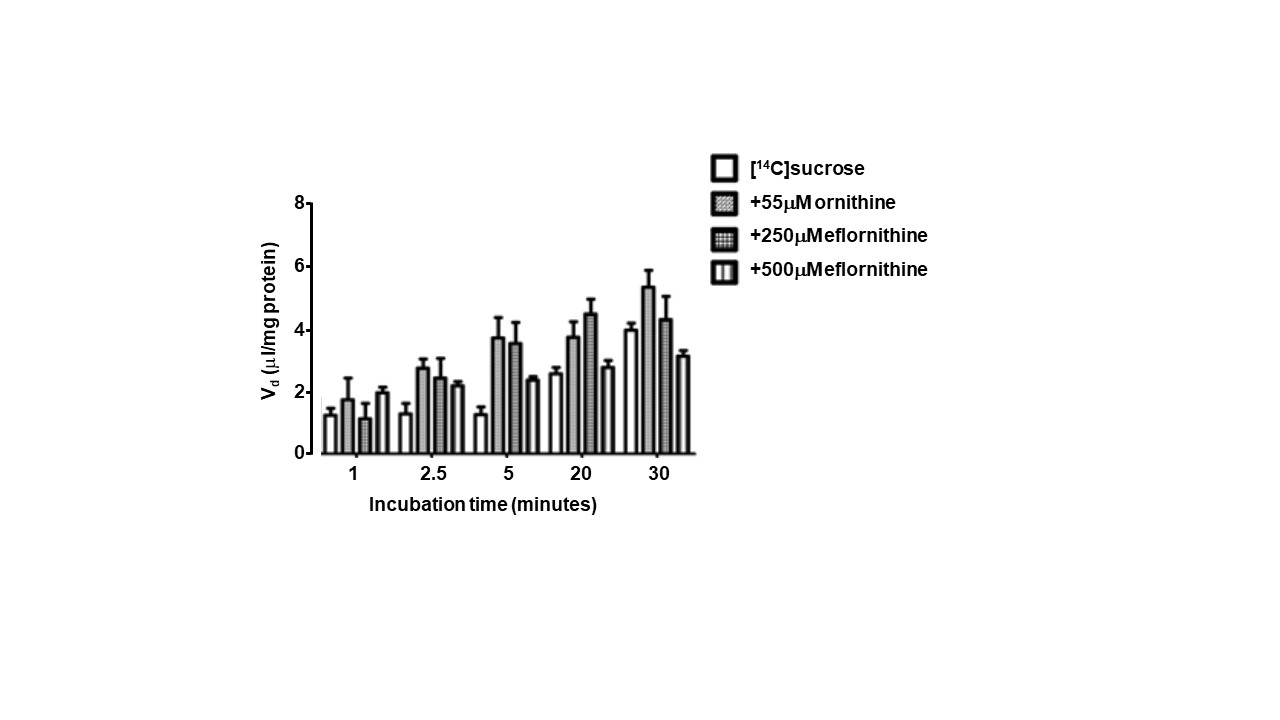

Supplement: Figure S3 [file EMS194562-supplement-Figure_S3.jpg]

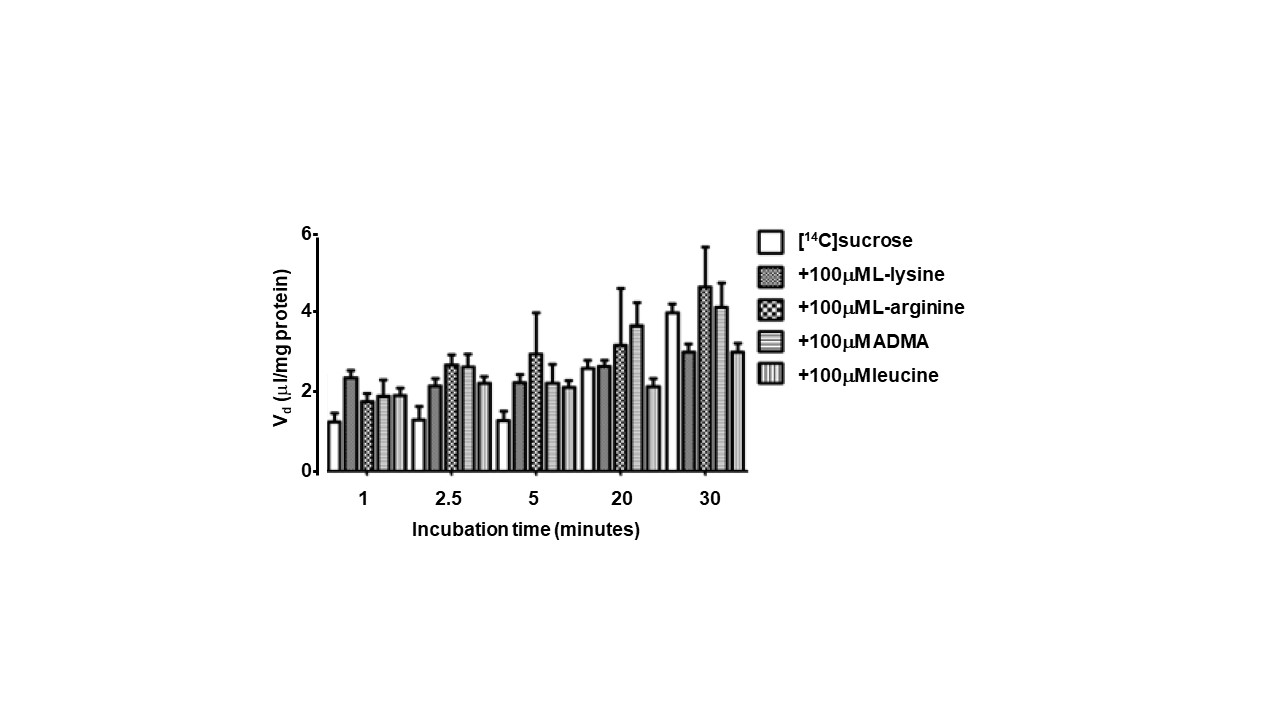

Supplement: Figure S4 [file EMS194562-supplement-Figure_S4.jpg]

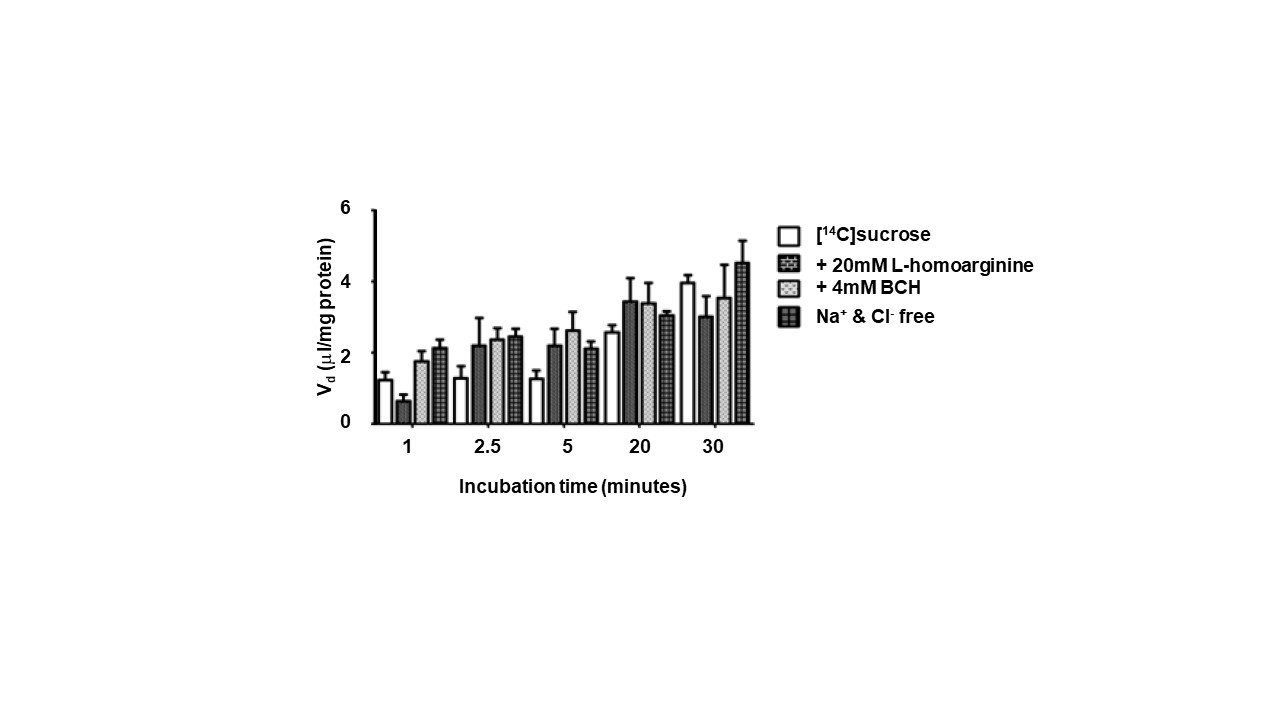

Supplement: Figure S5 [file EMS194562-supplement-Figure_S5.jpg]

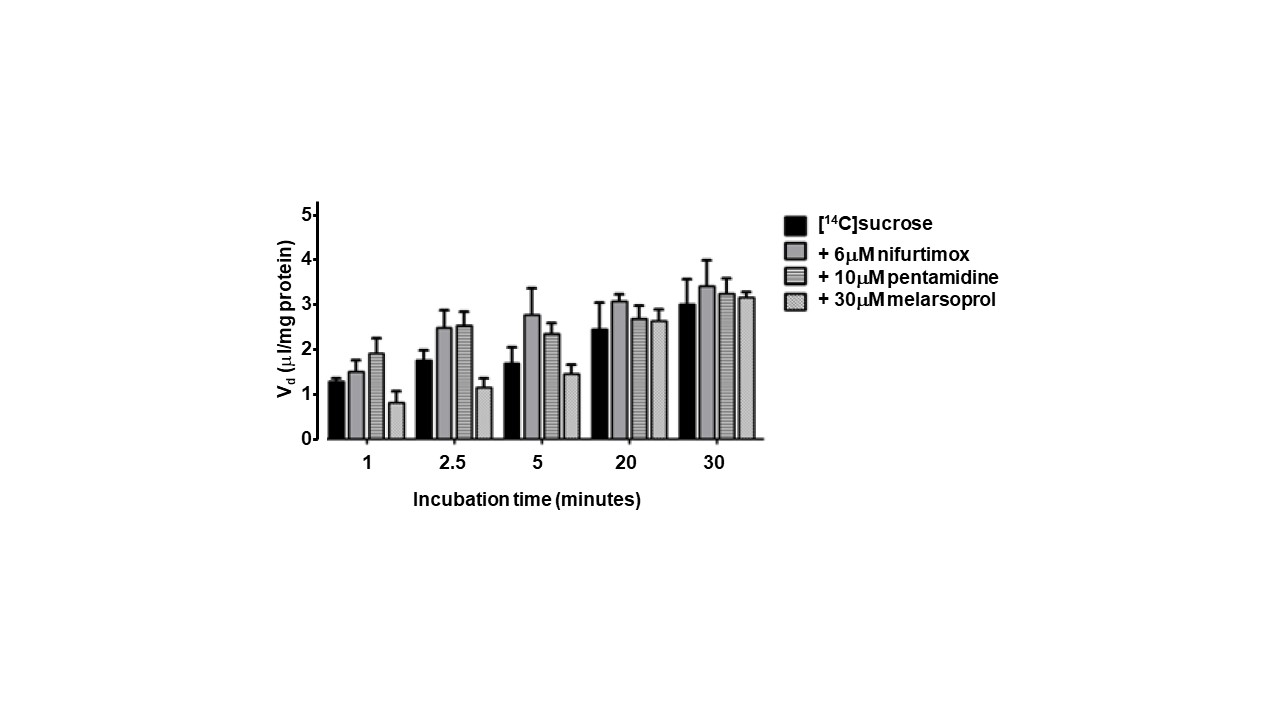

Supplement: Figure S6 [file EMS194562-supplement-Figure_S6.jpg]

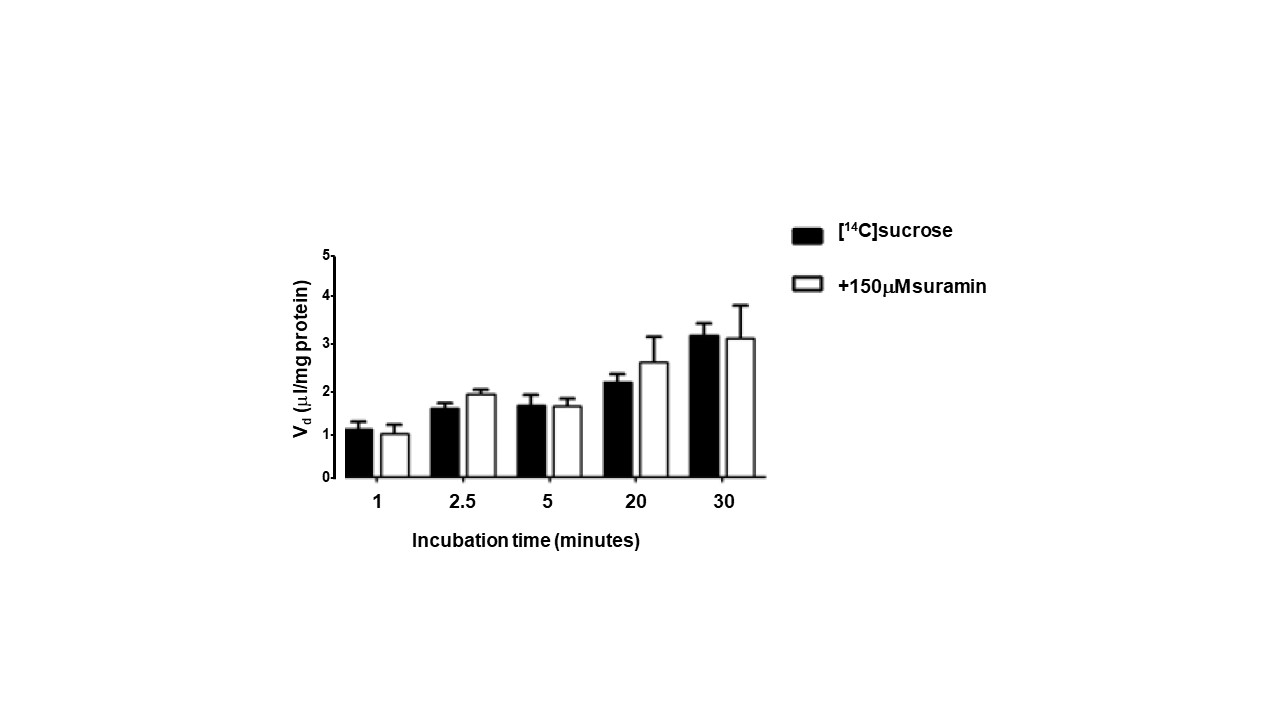

Supplement: Figure S7 [file EMS194562-supplement-Figure_S7.jpg]
