# Supplementary material for: Identification of transport systems involved in eflornithine delivery across the blood-brain barrier
Source: Front Drug Deliv. Author manuscript; Available in PMC 2024 Mar 13. (PMC7615738; doi:10.3389/fddev.2023.1113493)
Supplement: Table S1 [file EMS194562-supplement-Table_S1.docx]

Supplementary Material

Identification of transport systems involved in eflornithine delivery across the blood-brain barrier.

Christopher P. Watson^1^, Gayathri Nair Sekhar^1^ and Sarah A Thomas^1*^

*Corresponding author: [sarah.thomas@kcl.ac.uk](mailto:sarah.thomas@kcl.ac.uk)**Table S1:** Substrates/inhibitors used to identify CAA systems (White, 1985; Deves and Boyd, 1998; Closs, 2002; O’Kane et al., 2006; Watson et al., 2016). System y^+^ transporters are known as the cationic amino acid transporter (CAT) family and the transport systems, y^+^L, b^o,+^, and B^o,+^, are collectively known as the broad-scope amino acid transporter (BAT). The b^+^ system belongs to another family and has only been expressed in blastocysts (van Winkle and Campione, 1990). A complex of different proteins rather than a single carrier may mediate a distinct system activity and are also tabulated with their respective gene codes.

| *System* | *Transporter Protein* *Gene code* | *Na^+^ dependent* | *Substrates* | *Inhibitors* |
| --- | --- | --- | --- | --- |
| y^+^ | CAT1-3  SLC7A1-A3 | No  Yes | Lysine, Arginine, Ornithine, ADMA  Small NAA (weak) | *L*-Homoarginine |
| b^+^ | Not identified at molecular level | No | Arginine, Lysine.  NAA do NOT inhibit CAA transport even if Na^+^ present. | *L*-Homoarginine |
| b^0,+^ | rBAT/ b ^0,+^AT (SLC3A1/ SLC7A9) heteromeric complex | No  No | Lysine, Arginine,  Cystine, Leucine and other NAA | Harmaline |
| y^+^L | 4F2hc (SLC3A2) and y^+^LAT-1 (SLC7A7) or y^+^LAT-2 (SLC7A6) | No  Yes | Lysine, Arginine, Ornithine  Leucine, Methionine, Glutamine Other NAA | N-ethymaleimide  (NEM) |
| B^0,+^ | ATB^0,+^  SLC6A14 | Yes  Yes | Lysine, Arginine^(1)^, Alanine  Small and branched NAA | BCH |

1. Interestingly, the physiological relevance of the B^0,+^ system transporter protein, ATB^0,+^, in the transport of the cationic amino acid, arginine, is debated (Ahmadi et al., 2018; Fairweather et al., 2021).

**References**

Ahmadi, S., Xia, S., Wu, Y.-S., di Paola, M., Kissoon, R., Luk, C., et al. (2018). SLC6A14, an amino acid transporter, modifies the primary CF defect in fluid secretion. *Elife* 7. doi: 10.7554/eLife.37963.

Closs, E. I. (2002). Expression, regulation and function of carrier proteins for cationic amino acids. *Curr Opin Nephrol Hypertens* 11, 99–107. Available at: http://www.ncbi.nlm.nih.gov/pubmed/11753094 [Accessed March 25, 2016].

Deves, R., and Boyd, C. A. R. (1998). Transporters for Cationic Amino Acids in Animal Cells: Discovery, Structure, and Function. *Physiol Rev* 78, 487–545.

Fairweather, S. J., Okada, S., Gauthier-Coles, G., Javed, K., Bröer, A., and Bröer, S. (2021). A GC-MS/Single-Cell Method to Evaluate Membrane Transporter Substrate Specificity and Signaling. *Front Mol Biosci* 8. doi: 10.3389/fmolb.2021.646574.

MarvinSketch (2022). MarvinSketch. ChemAxon version 22.9.0. http://chemaxon.com Accessed November 2022.

O’Kane, R. L., Viña, J. R., Simpson, I., Zaragozá, R., Mokashi, A., and Hawkins, R. A. (2006). Cationic amino acid transport across the blood-brain barrier is mediated exclusively by system y+. *Am J Physiol Endocrinol Metab* 291, E412–E419.

van Winkle, L. J., and Campione, A. L. (1990). Functional changes in cation-preferring amino acid transport during development of preimplantation mouse conceptuses. *Biochim Biophys Acta* 1028, 165–73. doi: 10.1016/0005-2736(90)90151-d.

Watson, C. P., Pazarentzos, E., Fidanboylu, M., Padilla, B., Brown, R., and Thomas, S. A. (2016). The transporter and permeability interactions of asymmetric dimethylarginine (ADMA) and L-arginine with the human blood–brain barrier in vitro. *Brain Res* 1648. doi: 10.1016/j.brainres.2016.07.026.

White, M. F. (1985). The transport of cationic amino acids across the plasma membrane of mammalian cells. *Biochimica et Biophysica Acta (BBA) - Reviews on Biomembranes* 822, 355–374. doi: 10.1016/0304-4157(85)90015-2.
