# Supplementary material for: Identification of transport systems involved in eflornithine delivery across the blood-brain barrier
Source: Front Drug Deliv. Author manuscript; Available in PMC 2024 Mar 13. (PMC7615738; doi:10.3389/fddev.2023.1113493)
Supplement: Table S2 [file EMS194562-supplement-Table_S2.docx]

Supplementary Material

Identification of transport systems involved in eflornithine delivery across the blood-brain barrier.

Christopher P. Watson^1^, Gayathri Nair Sekhar^1^ and Sarah A Thomas^1*^

*Corresponding author: [sarah.thomas@kcl.ac.uk](mailto:sarah.thomas@kcl.ac.uk)

**TABLE S2**: A table listing the physicochemical characteristics for eflornithine and related substances. All values obtained from MarvinSketch (MarvinSketch, 2022) unless stated (e.g. DrugBank (Wishart et al., 2018)). The common name, the International Union of Pure and Applied Chemistry (IUPAC) name and the chemical abstracts service (CAS) registry number have been reported for each substance.

| **Common Name**  **IUPAC NAME** | **CAS number** | **MW**  **LogD**  **at pH 7.4** | **Gross charge distribution of the molecule at pH 7.4** | **Physiological charge of major microspecies**  **Drug Bank** | **% Distribution of microspecies (# or macrospecies) at pH 7.4 together with their charge.** |
| --- | --- | --- | --- | --- | --- |
| **B^0,+^ system inhibitor** |  |  |  |  |  |
| BCH  2-aminobicyclo-(2,2,1)-heptane-2-carboxylic acid | 20448-79-7 | 155.20  -2.50 | -0.003 | Not available. | (0) 99.67%  (-1) 0.33% |
| **Anti-HAT Drugs** |  |  |  |  |  |
| Pentamidine  4-{[5-(4-carbamimidoylphenoxy)pentyl]oxy}benzene-1-carboximidamide | [100-33-4](https://commonchemistry.cas.org/detail?cas_rn=100-33-4) | 340.42  -2.84 | +2.00 | +2 | +2 (99.9%)  +1 (0.01%) |
| Melarsoprol  (2-{4-[(4,6-diamino-1,3,5-triazin-2-yl)amino]phenyl}-1,3,2-dithiarsolan-4-yl)methanol | [494-79-1](https://commonchemistry.cas.org/detail?cas_rn=494-79-1) | 398.33  0.56 | 0.840 | +1 | (+1) 76.24%  (0) 14.51%  (+1) 9.25% |
| Nifurtimox  3-methyl-4-[(E)-[(5-nitrofuran-2-yl)methylidene]amino]-1lambda6-thiomorpholine-1,1-dione | 23256-30-6 | 287.29  -0.08 | 0.00 | 0 | 1. 100 % |
| Suramin  8-{4-methyl-3-[3-({[3-({2-methyl-5-[(4,6,8-trisulfonaphthalen-1-yl)carbamoyl]phenyl}carbamoyl)phenyl]carbamoyl}amino)benzamido]benzamido}naphthalene-1,3,5-trisulfonic acid | [145-63-1](https://commonchemistry.cas.org/detail?cas_rn=145-63-1) | 1297.28  -10.10 | -6.000 | -6 | #(-6) 99.9% |

**Reference**

Wishart, D. S., Feunang, Y. D., Guo, A. C., Lo, E. J., Marcu, A., Grant, J. R., et al. (2018). DrugBank 5.0: a major update to the DrugBank database for 2018. *Nucleic Acids Res* 46, D1074–D1082. doi: 10.1093/nar/gkx1037.
