# Supplementary material for: Identification of transport systems involved in eflornithine delivery across the blood-brain barrier
Source: Front Drug Deliv. Author manuscript; Available in PMC 2024 Mar 13. (PMC7615738; doi:10.3389/fddev.2023.1113493)
Supplement: Supplementary Figure Legend [file EMS194562-supplement-Supplementary_Figure_Legend.pdf]

secondary antibody and viewed at 63x with oil emersion using a Zeiss LSM710 confocal microscope and image analysis software Zen 2009. Scale bar 10 μm. Cell nuclei were counterstained with 1 mg/ml DAPI. For negative staining, cells were stained with secondary antibody only along with DAPI (inset figures).

Figure S1: The chemical structure of the major microspecies of eflornithine and other amino acids at physiological pH.

Figure S2: Line graphs showing the volume of distribution of [^{3}H]eflornithine and [^{14}C]sucrose into hCMEC/D3 cells plotted as a function of incubation time. The V_d of [^{3}H]eflornithine has not been corrected for [^{14}C]sucrose. All data expressed as mean ± S.E.M, n = 3-4 (plates), with 6 replicates (wells) per plate. Linear regression analysis of the data determined the lines of best fit which are shown as dashed lines. The rate of accumulation over 30 minutes can be determined as the slope (m) of the computed regression lines and is reported together with the ordinate intercept (c) and the coefficient of determination or R^2 -value. The ordinate intercept represents the rapidly equilibrating space which includes the endothelial cell space and non-specific binding to both the cell membranes and plasticware.

Figure S3: The effect of unlabelled eflornithine and ornithine on the accumulation of [^{14}C]sucrose in hCMEC/D3 cells. All data expressed as mean ± S.E.M, n = 3-5 (plates), with 6 replicates (wells) per plate. Data were analysed using two-way ANOVA with SigmaPlot 13. No significant differences compared to control were observed.

Figure S4: The effect of unlabelled L-lysine, L-arginine, ADMA or leucine on [^{14}C]sucrose accumulation in hCMEC/D3 cells. Significant differences compared to control were not observed in hCMEC/D3 cells. All data expressed as mean ± S.E.M, n = 3-4 (plates), with 6 replicates (wells) per plate. Data were analysed using two-way ANOVA with SigmaPlot 13.

Figure S5: [^{14}C]sucrose accumulation in hCMEC/D3s in the absence and presence of L-homoarginine, BCH or Na^+Cl^- free buffer. All data expressed as mean ± S.E.M, n = 3 (plates), with 6 replicates (wells) per plate. Data were analysed using two-way ANOVA with SigmaPlot 13. No significant differences compared to control were observed at all time-points.

Figure S6: The effect of anti-HAT drugs on [^{14}C]sucrose accumulation in hCMEC/D3 in the presence of DMSO. All data expressed as mean ± S.E.M, n = 3 (plates), with 6 replicates (wells) per plate. Data were analysed using two-way ANOVA with SigmaPlot 13. No significant differences were observed in hCMEC/D3 cells. The control and test groups were performed in the presence of 0.05% DMSO.

Figure S7: The effect of suramin on [^{14}C]sucrose accumulation in hCMEC/D3s. All data expressed as mean ± S.E.M, n = 4 plates, with 6 replicates (wells) per plate. Data were

analysed using two-way ANOVA with SigmaPlot 13. No differences compared to control were observed.
